# Negotiation of new international health law on intellectual property, technology transfer, open science and pathogen access and benefit sharing: a textual and contextual analysis

**DOI:** 10.1136/bmjph-2024-001467

**Published:** 2024-12-17

**Authors:** Jeremy de Beer, Rosa De Koker

**Affiliations:** 1 Faculty of Law, University of Ottawa, Ottawa, Ontario, Canada; 2 College of Business and Economics, University of Johannesburg, Johannesburg, South Africa; 3 Student at the University of Ottawa, Ottawa, Ontario, Canada

**Keywords:** legislation and jurisprudence, Public Health, COVID-19, Vaccination

## Abstract

**Introduction:**

This original research article reframes new international law and policy developments at the intersections of open science, technology transfer, intellectual property and access and sharing the benefits of biomedical innovation in the context of global pandemics.

**Methods:**

Through textual and contextual analysis, using the lens of national experiences and negotiating positions of one high-income country, Canada, it traces the evolution of legal normative developments from a proposed waiver of aspects of the World Trade Organisation Agreement on Trade-Related Aspects of Intellectual Property Rights through a forum shift to negotiate a new agreement for pandemic preparedness and response (the Pandemic Agreement) at the WHO.

**Results:**

The analysis shows a significant evolution of treaty provisions on R&D, sustainable and diversified production of pandemic-related products, transfer of technology and know-how and access and benefit sharing. Juxtaposed against a Canadian parliamentary report, stakeholder consultations and Canada’s stated commitment to equity, the negotiating texts reflect major compromises on the key issues. High-income countries sought open access to pathogen gene sequence data crucial for pandemic prevention, preparedness and response while resisting measures other than voluntarily licensing on mutually agreed terms of private intellectual property rights derived from the use of such data or other publicly funded research. Low- and middle-income countries, meanwhile, were offered diluted language on technology transfer in exchange for a new pathogen access and benefit-sharing scheme.

**Conclusions:**

The practical effects of these compromises remain to be seen, but they risk exacerbating rather than ameliorating global health inequities.

**WHAT IS ALREADY KNOWN ON THIS TOPIC**

- Extensive research shows that intellectual property rights impact access to medicines and explores tensions among economic incentives for innovation and outcomes for global health. Few studies show how exactly these tensions manifest in real-time negotiations of new international law and policy instruments, such as the WHO’s Pandemic Agreement. While there is an emerging awareness of the newest international health law instrument, the Pandemic Agreement, little is widely known about intellectual property-related provisions and their links with open science, technology transfer and access and benefit sharing.

**WHAT THIS STUDY ADDS**

- This study adds new knowledge about the latest legal normative developments in global health and how they happened by documenting the shift in negotiations from trade law to health law forums and the evolving draft text of the latest international legal provisions pertaining to open science, technology transfer, intellectual property and access to and sharing the benefits of biomedical innovation. By focusing on Canada’s role and position, the study adds depth and specificity to the analysis of global negotiations while also offering insights relevant to the global health community.

**HOW THIS STUDY MIGHT AFFECT RESEARCH, PRACTICE OR POLICY**

- Seeing how forum shifting and negotiation strategies lead to compromises in international health law and policymaking enables a better understanding of emerging legal rules and their likely practical impacts, facilitates more effective national implementation of international law and supports further efforts to promote equity in global health.

## Introduction

 Research establishes that intellectual property (IP) law can exacerbate global and domestic health inequalities by contributing, in part, to unequal access to vaccines.^1 2^ Discussion of a potential ‘Trade-Related Aspects of Intellectual Property Rights (TRIPS) waiver’ at the World Trade Organisation (WTO) during the COVID-19 pandemic built upon extensive earlier work on IP and access to medicines, such as antiretrovirals to fight HIV.^3^ The issue of IP and vaccine access has often been framed from the perspective of low-income and middle-income countries, or LMICs for short, sometimes referenced as less-developed countries.^4^,^6^ The Director of the WHO labelled the inequality between high- and low-income countries as ‘vaccine apartheid’.^7^,^9^ The inequalities caused by COVID-19 are determined not only by a country’s income level but also by an individual’s gender.^10^,^12^ Factors that make vaccine-related barriers to technology transfer ‘a feminist issue’^13^ include the under-representation of women in science and technology and the well-documented gender gaps in patenting and IP ownership.^14 15^

Inequitable impacts of IP-related barriers to access to medicines, specifically gender-related inequalities, have also been demonstrated in high-income countries.^16^ Access inequalities in high-income countries have prompted calls for those countries to limit or override domestic IP rights in certain situations.^17 18^ It has also been suggested that the experience with COVID-19 vaccines should trigger restructuring of innovation systems by, for example, nuancing Canada’s approach to IP and accelerating Canada’s global contributions through open science.^19^ The Pandemic Agreement presented a concrete chance to advance those aims. In this respect, Canada stands as a proxy for other high-income countries that, lacking domestic biomanufacturing capacity and being net importers of IP, might aim to reduce IP-related access barriers.

Canada’s role specifically in global vaccine equity failures has been well documented in recent research^20 21^ and concerns have been expressed in policy commentary specifically about Canada’s positions on the WHO Pandemic Agreement.^22^ This study builds on the latest collection of analyses on accountability for Canada’s COVID-19 response^23^ with new research tracking interventions at the Intergovernmental Negotiating Body (INB) tasked to draft and negotiate the Agreement. It answers the invitation for ‘a deeper analysis of the discursive dynamics’ of ‘dissonances between Canada’s rhetoric on vaccines and actions concerning vaccine and health equity on the global stage’.^20^ For example, despite Canada’s stated commitment to promote gender equality, the Canadian position is likely to exacerbate gender disparities given the specific contexts here, that is, a context where women are significantly under-represented as owners of IP and technology ventures and yet also disproportionately impacted by the harmful impacts of inequitable access to medicines.

The article begins with a discussion of the TRIPS waiver and explores—through the report of the House of Commons Standing Committee on Foreign Affairs and International Development, *Overcoming the Barriers to Global Vaccine Equity and Ending the Pandemic*^24^—how it was initially perceived by parliamentarians and stakeholders. The subsequent sections of the article, recognising the transition in attention from the WTO TRIPS waiver to the development of the WHO Pandemic Agreement, detail the evolution of the Pandemic Agreement’s IP-relevant articles, how Canada publicly engaged stakeholders regarding the issues and the dissonance between elected parliamentarians’ recommendations, stakeholders’ comments, the government’s public stance and its ultimate negotiating positions. The study shows how those perspectives on IP contributed to shaping the proposed text of international law provisions addressing IP-related issues linked to open science, technology transfer and pathogen access and benefit sharing.

## Methods

The study is based on a desk review of legal and policy materials. We closely followed the processes of the INB, immediately reviewing and analysing officially released negotiating texts. We monitored (informally, not systematically) news and social media commentary to glean insights on national (specifically Canadian) negotiating positions, including via unofficial releases of draft negotiating texts with countries’ proposed markups. The official and unofficial negotiating texts analysed in this study are listed in table 1. We also followed all official government of Canada updates and newsletters and participated actively in the government’s public consultations. Based on these legal and policy materials, we applied legal doctrinal research methods, including descriptive qualitative and legal normative analysis.

**Table 1:** Official and unofficial texts of the WHO Pandemic Agreement

Date	Document ID	Title
16 September 2024	A/77/10	Proposal for the WHO Pandemic Agreement (draft text reflecting progress up to Monday 16 September at 17:30 CEST)
24 May 2024	A/77/10 Annex	Proposal for the WHO Pandemic Agreement (draft text reflecting progress up to Friday 24 May at 12:00 CEST)
22 April 2024	A/INB/9/3 Rev.1	Proposal for the WHO Pandemic Agreement
27 March 2024	A/INB/9/3 (Markup)	Revised draft of the negotiating text of the WHO Pandemic Agreement (ONSCREEN TEXT)
13 March 2024	A/INB/9/3	Revised draft of the negotiating text of the WHO Pandemic Agreement
30 October 2023	A/INB/7/3	Proposal for negotiating text of the WHO Pandemic Agreement
2 June 2023	A/INB/5/6	Bureau’s text of the WHO convention, agreement or other international instrument on pandemic prevention, preparedness and response (WHO CA+)
22 May 2023	A/INB/X/X (Advance)	DRAFT Bureau’s text of the WHO CA+ (ADVANCE COPY, UNEDITED)
4 April 2023	A/INB/4/3 (Markup)	Zero draft of the WHO CA+for the consideration of the Intergovernmental Negotiating Body at its fourth meeting (ONSCREEN TEXT)
1 February 2023	A/INB/4/3	Zero draft of the WHO CA+for the consideration of the Intergovernmental Negotiating Body at its fourth meeting
25 November 2022	A/INB/3/3	Conceptual zero draft for the consideration of the Intergovernmental Negotiating Body at its third meeting

## Findings, discussion and analysis

### The TRIPS waiver

Legally, the ‘TRIPS waiver’ is not really a waiver of patents or IP protection.^25^ Rather, it is a waiver of WTO member states’ international trade law obligations in respect of IP rights. Waiving WTO member states’ international legal obligations allows those member states to subsequently implement flexibilities, limitations and exceptions to IP rights into their respective national laws. Those national flexibilities would then have the effect of altering the legal scope of privately held IP rights in the specific territories taking advantage of the waiver of international legal obligations.

Originally proposed by India and South Africa in October 2020, the initial vision of the TRIPS waiver tabled at the WTO^26^ was to increase access to all COVID-19-related medical technology by allowing widespread use of essential manufacturing information.^27^ In December 2020, Global Affairs Canada, in a statement linked to the WTO TRIPS waiver, wrote that:

Canada’s longstanding view is that IP rights can serve as an important incentive to innovation, while ensuring that there is an appropriate balance between protecting IP rights and promoting access to medicines and other healthcare technologies.^28^

This early statement foreshadowed Canada’s refusal to support the WTO TRIPS waiver and its subsequent negotiating positions around the WHO Pandemic Agreement.

The WTO operates via a consensus-based decision-making process, and thus, throughout many months of negotiation, the proposal evolved in content and form.^27^ The eventual ministerial decision, adopted on 17 June 2022, restricted the waiving of IP rights to vaccines, with a time limit of 5 years.^29^ The decision pertained to Article 31(f) of the TRIPS Agreement, which requires the use of the flexibilities provided for under Article 31 to be predominately for the supply of its domestic markets. Flexibilities provided for under Article 31 of the TRIPS Agreement have faced criticism for being onerous and unusable, and thus, the narrow exception the TRIPS waiver provided was also critiqued as inadequate. ‘It is hard to imagine anything with fewer benefits’, writes one expert about the WTO decision.^30^ Another reflects, ‘at some point, one has to wonder what capabilities the WTO still has to facilitate the development of effective responses to future global and regional crises’.^31^

Earlier in 2022, Canada’s House of Commons Standing Committee on Foreign Affairs and International Development began a study of the government’s involvement in addressing global vaccine inequities and included an investigation of the role of IP. Composed of 12 Members of Parliament, the committee acknowledged part of the study was meant to ‘scrutinise the efforts [of] the Canadian government’ in failing to meaningfully help the WHO meet world vaccination rate goals.^24^ The primary topic of scrutiny was directed towards IP, most specifically in relation to the Canadian position on the TRIPS waiver.^24^

In its October 2022 report, many of the committee’s various witnesses who provided evidence to the committee reiterated sentiments expressed in Canadian media, namely the frustration, confusion and disappointment in Canada’s lack of a position on the originally proposed TRIPS waiver.^32^,^34^ For example, one witness explained that while the Canadian government never outwardly rejected the waiver, it also failed to engage on any specifics bringing the waiver to realisation.^24^ Another expert suggested that Canada ‘kicked the can down the road’ until the watered-down waiver was finalised.^24^ Civil society representatives had called on Canada to support a more comprehensive waiver that would have included ‘all necessary medical technologies’ and been open to all countries ‘with a means to contribute to vaccine production’.^24^

Pharmaceutical industry representatives stated IP was a ‘false problem’, rejecting the notion that IP reduced equitable access to COVID-19 vaccines.^24^ Government emails and documents released to the parliamentary committee showed close ties between the Canadian government and pharmaceutical companies, with industry representatives reportedly praising the government’s ‘muted response’ to the TRIPS waiver.^35^

A senior official responsible for health and nutrition at the Department of Foreign Affairs, Trade and Development emphasised that the lack of vaccination rates in LMICs had to do with ‘health system capacity’ and ‘misinformation’, rather than any supply shortage that the waiver would mitigate.^24^ The Minister of International Trade, Export Promotion, Small Business and Economic Development herself had similarly stated, in May 2022, that many barriers to vaccine equity ‘are unrelated to IP’ and that the government ‘firmly’ believes in protecting IP.^36^ The minister recognised ‘the integral role that industry has played in innovating’ COVID-19 vaccines, attempting to explain why Canada was not an active supporter of the original TRIPS waiver.^36^

Based on the evidence collected during months of hearing from various stakeholders, the parliamentary report included 11 recommendations for the Canadian government in alleviating vaccine inequities.^24^ The report specifically recommended ‘that the Government of Canada advocate for an extension of the June 2022 Ministerial Decision on the TRIPS Agreement to cover the production and supply of COVID-19 diagnostics and therapeutics’.^24^ The report also recommended immediately amending Canadian domestic law to allow processes of compulsory licensing for the export of vaccines, diagnostics and treatments for COVID-19 to at least commence, which the government had not been willing to do during the pandemic.^24^

The Canadian government’s official response to the committee report, presented to the House of Commons on 6 March 2023, ‘took note’ of the report’s recommendation to extend the TRIPS waiver but maintained a non-committal approach.^37^ The government acknowledged the WTO General Council would revisit the topic soon thereafter, and, in the meantime, Canada would encourage ‘evidence-based’ exchanges as to whether or not the supply of COVID-19 diagnostics and therapeutics is impaired by the TRIPS agreement.^37^ Thus, as other researchers have noted, it appeared as though the Canadian government never outwardly rejected the waiver but also never actively worked towards its realisation.^21^

By March 2023, the WTO had confirmed that any decision to extend the TRIPS waiver to therapeutics and diagnostics had been ‘pushed off’, citing the continued ‘elusive’ consensus among WTO members.^38^ Developing and least developed country (LDC) members expressed concern that a solution for vaccines alone is insufficient, while according to the WTO, ‘other members’ (ie, Canada) continued to advocate for ‘more evidence and fact-based discussions’ and questioned ‘whether an IP-induced access problem existed’.^38^

Considering that the final text of the TRIPS waiver failed to address the impacts of trade secrets, copyright, industrial design and technical know-how, the sufficiency of the waiver to deal with the complexities involved in vaccine manufacturing was always questionable.^39^ In light of developments since the height of the COVID-19 pandemic, it seems unlikely the TRIPS waiver will ever be extended to therapeutics and diagnostics.^40^

Meanwhile, the world’s attention turned to the negotiation of what has come to be known as the WHO Pandemic Agreement. In the following sections, we examine the development of some of the key IP-related provisions in the Pandemic Agreement from its inception as a conceptual zero draft in 2021, through proposed negotiating texts in 2023, to final draft texts in 2024. Our analysis primarily focuses on the development of the key article on technology transfer and know-how, while also tracking provisions on research and development (R&D), sustainable production and access and benefit sharing.

### The Pandemic Agreement

#### The ‘zero’ draft from November 2022 to February 2023

194 WHO members states agreed in December 2021 to launch the International Negotiating Body (INB) to develop a Pandemic Agreement.^41^ Soon after, an international expert group on IP and access to medicines had already developed and published recommendations for the instrument’s to-be-negotiated provisions on IP and technology.^42^ Among such recommendations were creating conditions for licensing government-funded R&D, mandating technology transfer and sharing IP, data and knowledge. The recommendations noted the importance of various forms of IP, including patents, undisclosed information (know-how) and test data.^42^ Meanwhile, other researchers drew policymakers’ attention to the need for international copyright flexibilities, for example, to access relevant research publications or to mine relevant text and data for computational research.^43^ Such analyses helped to identify some of the key issues and establish a framework for drafting a proposed text.

In November 2022, the INB produced a conceptual ‘zero’ draft for the Agreement. The conceptual zero draft recognised ‘equity should be a principle, an indicator and an outcome of pandemic prevention, preparedness and response’.^44^ This zero draft evolved slightly in structure and content from its release in November 2022 for the INB’s third meeting to its re-release in February 2023 for the INB’s fourth meeting, as detailed below.

To increase research and development capacities, the conceptual zero draft called for parties to recognise the need for ‘open science approaches for rapid sharing of scientific findings and research results’.^44^ Another key provision aimed to improve access to technology by ‘promoting sustainable and equitably distributed production and transfer of technology and know-how’.^44^ To achieve that aim, the November 2022 draft proposed that parties would ‘bolster and strengthen…regulatory authorities’ capacities, to prepare for and accelerate emergency licensing and approval procedures…to allow for the timely availability of essential pandemic response products’.^44^ That language was retained but moved to a different article by February 2023, when the zero draft was released. The February 2023 draft also suggested that parties shall ‘encourage entities…that conduct research and development of prepandemic and pandemic products…to grant, on mutually agreed terms, licences to capable manufacturers…to use their intellectual property…’.^44^

The zero drafts also addressed the topic of compulsory licensing and states’ international trade law obligations, that is, the essence of the earlier and then-still-ongoing TRIPS waiver discussions. The November 2022 conceptual draft proposed that each party ‘[shall]/[should]’ strengthen local capacity to manufacture pandemic response products through the transfer of technology and know-how by means that include ‘…measures to support time-bound waivers of protection of intellectual property rights…’.^44^ By February 2023, the language had been slightly strengthened to state that the parties ‘will take appropriate measures to support time-bound waivers of intellectual property rights…to the extent necessary to increase the availability and adequacy of affordable pandemic-related products’.^45^ The provision continued to require that parties ‘apply the full use of the flexibilities provided in the TRIPS Agreement’, ‘shall encourage all holders of patents…to waive or manage as appropriate, payment of royalties by developing country manufacturers’, and ‘shall encourage all research and development institutes, in particular those receiving significant public financing’ to also waive or manage royalties.^45^

#### Canadian Stakeholder Engagement March 2023

Just over a month after the release of the zero draft, around 200 representatives from across Canada’s provinces and territories were invited by the government of Canada to take part in the *Pandemic Instrument Partner and Stakeholder Engagement Forum* hosted by the Office of International Affairs for the Health Portfolio.^46^ The forum aimed to help inform Canada’s priorities and objectives in relation to the then-upcoming pandemic treaty negotiations.^46^ The forum was composed of various stakeholders, including non-governmental organisations (NGOs), academics, youth, industry representatives and members of the public and private sector.^47^ Following the forum, an official report summarising the findings was released by the Government of Canada, which we briefly summarise below.

One of the six themes of discussion was ‘equitable access to pandemic response products’. Participants explored how the pandemic instrument could be leveraged to provide equitable access to pandemic response products.^47^ They recommended that the Agreement include specific language allowing access to products and knowledge and identified the importance of supporting technology transfer, which specifically includes ‘providing IP rights waivers [which] can help accelerate the availability and distribution of pandemic response products, ensuring equitable access globally’.^47^ To avoid the inequitable distribution of vaccines that the world experienced during the COVID-19 pandemic, vaccine sharing, technology transfer and financial support obligations were commitments that participants wished to see Canada fulfil.^47^

Participants also took part in roundtable discussions. The Public Health Agency of Canada reported that academics, experts and researchers wished to see the Pandemic Agreement ensure that there is a ‘focus on…sharing IP, not economic interests of private companies’ to facilitate equitable access to pandemic products.^47^ Stakeholders also expressed that the Agreement should implement specific language on how IP waivers will be implemented, such as mechanisms for compulsory licensing and technology transfer.^47^

International civil society organisations submitted the Pandemic Agreement should balance the reality that patents play a role in sparking innovation while also acknowledging and preventing excessive profits.^47^ Additionally, time-bound patent waivers ‘should be explored’ and IP flexibility should be advocated for in times of emergency.^47^ Representatives of private sector businesses, specifically pharmaceutical companies, submitted that the zero draft contained ‘problematic’ language in relation to IP infringement and the TRIPS waiver, that innovation depends on strong IP protection and that the WTO should handle IP considerations.^47^

The engagement forum highlighted a variety of Canadian voices and opinions, of which the underpinning of all discussions surrounded how ‘equity must be a primary consideration’ in the creation and development of the instrument.^47^ How the above stakeholder perspectives were, or were not, incorporated into the subsequent drafts and negotiating texts is explored below.

#### April 2023 annotations on the zero draft

During an INB drafting group meeting in April 2023, shown onscreen was an annotated markup of the zero draft with specific language proposed by different WTO member states. The use of square-bracketed text to indicate suggested textual additions or deletions is a normal way that international legal instruments are negotiated. Also increasingly common is the unofficial release and online circulation of these annotated square-bracket texts, which is what happened when Politico published the INB drafting group’s 4 April 2023 markup of the zero draft.^45^ This is the first time that observers and researchers worldwide were able to analyse the negotiating positions of each country via specific textual suggestions.

Canada’s suggestions focused on ensuring all measures in relation to technology transfer, including know-how, data and pathogen sample sharing, are ‘voluntary’ and on ‘mutually agreed terms’.^45^ This aligns with a statement by the Canadian government responding to its domestic parliamentary report: ‘public benefit is maximised when IP terms and conditions are tailored to address the specific context and objective of each funding opportunity on voluntary, mutually agreed terms’.^37^

In addition to Canada’s reservations on the guiding principles, the new article on technology transfer sparked attention.^45^ Annotations show Canada proposing to add ‘voluntary’ directly into the title of the article, in addition to various provisions thereunder.^45^ Canada also sought to add that in remedying inequitable access to pandemic-related products, ‘promoting investments’ should be addressed.^45^ Proposed additional language that would limit technology transfer in interpandemic times to situations where it is ‘appropriate’ and ‘feasible’.^45^ Canada suggested deleting the draft article supporting IP waivers, replacing that with reliance on existing flexibilities in the TRIPS agreement.^45^

While generally advocating for the weakening of the IP language, Canada also advocated to consider gender equity in the text. For example, the annotated text shows Canada requesting the following provision be added: ‘it recognises that pandemics have a disproportionate impact on women and girls, Indigenous peoples and all marginalised groups or people in vulnerable situations’.^45^ Canada’s suggestions, however, ignored gender-based aspects of IP protection.

#### The June 2023 first draft

After five official INB meetings and multiple drafting sessions during which the zero draft developed, the ‘first draft’ text was released in June 2023. In the June text, the most notable IP-related provisions were in Article 11 on technology transfer, which was presented in two different forms, options 11.A and 11.B.

Option 11.A read that during pandemic times, parties shall ‘take appropriate measures to support time-bound waivers of intellectual property rights that can accelerate or scale up the manufacturing of pandemic-related products during a pandemic, to the extent necessary to increase the availability and adequacy of affordable pandemic-related products’.^48^ Option 11.A further indicated that during pandemic times, existing TRIPS flexibilities shall be applied to their full use and that parties shall encourage the waiving of royalties on pandemic-related product patents and technology. This option seemed to align with Canadian civil society and academics’ contributions to the Engagement Forum, whereby participants advocated for more data and information sharing and a flexible approach to IP protection during pandemic times.

Option 11.B, stressed the ‘voluntary transfer of technology on mutually agreed terms as appropriate’^48^ and contained further division within its provisions, with options to completely eliminate any mention of TRIPS flexibilities and time-bound IP waivers. This option appeared in line with the views of Canadian pharmaceutical industry stakeholders, which the Canadian government seems to have adopted and implemented via Canada’s official textual suggestions. The removal of these provisions contradicted one of the main takeaways from the Canadian Stakeholder Engagement Forum: ‘The instrument must promote the sharing of information, data, best practices, expertise and resources’.^47^

The draft text had critics suggesting the instrument was being ‘watered down’ based on the removal of certain provisions and weakened language.^49 50^ For example, the term ‘as appropriate’ became associated with technology transfer provisions, with the February zero draft mentioning the term three times,^51^ compared with the June text, which mentioned the term ‘as appropriate’ six times.^48^ This language was influenced by Canada and other high-income countries, as shown in the April annotated draft.^45^

Some changes were well received by NGOs, such as the addition of a clause requiring countries to share genomic sequencing data.^49^ Largely unexplored, however, was the double standard that had been introduced with such a clause. While high-income countries proposed diluting language that would require any mandatory sharing of technology, know-how or data, they simultaneously proposed that all countries be obliged to share the gene sequence data on which technological innovation is based. This proposed mandatory sharing of innovation inputs, but voluntary sharing of innovation outputs, became a turning point in the negotiation process.

#### October 2023 negotiating text

Preparing for the seventh INB meeting that was held in early November 2023, a proposed negotiating text was presented to WHO member states in October 2023.^52 53^ Articles 10 and 11 on research and development and technology transfer of the October negotiating text contained most IP-related provisions.

Article 11 on technology transfer adopted a modified version of option 11.A of the June text, with significant edits and a new Article 10 on sustainable production. Acknowledgement of the relationship between inequitable access to pandemic products and manufacturing capacity was removed from the technology transfer article and was added to the new sustainable production article. Option 11.A of the June draft text stated that ‘inequitable access to pandemic-related products (including but not limited to vaccines, therapeutics and diagnostics) should be addressed by increased manufacturing capacity that is more equitably, geographically and strategically distributed’.^48^ This acknowledgement remained in part in the October text, but it removed mention of ‘vaccines, therapeutics and diagnostics’ and clarified that the point of addressing such inequity is to ‘reduc[e] the potential gap between supply and demand at the time of a pandemic’.^53^

Provisions related to strengthening and strategically locating manufacturing capacity, applying the full use of the flexibilities provided in the TRIPS Agreement and encouraging entities receiving public funding to grant licenses to manufacturers in developing countries to use IP were moved into the new Article 10 on sustainable production. It added that licenses are merely encouraged to be granted for IP use (especially for those that have received significant public financing) and are ‘non-exclusive, royalty-free licenses’.

Article 11 on technology transfer became increasingly contested for including more explicit language.^54^ For example, it stipulated that ‘each Party shall… commit to agree upon…time bound waivers of intellectual property rights to accelerate or scale up the manufacturing of pandemic-related products’.^53^ Finally, adopting parts of option 11.B of the June text, the October negotiating text added reference to the promotion of voluntary licensing within its article on sustainable production; however, language that encouraged the licenses to be granted ‘at the earliest opportunity and to the fullest extent possible’ was removed.^53^

Canada’s representative at the seventh INB forum commented that ‘Canada strongly supports advancing health equity’ through the Agreement.^52^ The comments focused on how the Agreement should ‘strengthen inclusive and gender-responsive health systems’, but that the Agreement was ultimately failing to do so.^52^ Furthermore, the comments praised the new text for strengthening provisions on multisectoral data and confirmed Canada believes ‘facilitating more effective data sharing among and between member states will be crucial at all points in the pandemic cycle’.^52^

#### February 2024 Canadian Stakeholder Consultations

In December 2023, the Canadian government sent re-engagement emails to partners and stakeholders that participated in the March 2023 Pandemic Instrument Stakeholder Engagement Forum.^55^ Participants who reconfirmed their interest in the process received an update in late December 2023 with a newsletter of updates on recent INB meetings and plans for future engagement. The newsletter announced there would be a Pandemic Instrument Partner and Stakeholder Regional Engagement Series, composed of half-day multistakeholder discussions in several Canadian cities (both in-person and virtual).^56^ The Engagement Series also included half-day working group meetings in three cities on ‘Canada’s three lean-in issues’, one of which was Health Equity.^56^ The newsletter confirmed the engagement series would build on the momentum from the March forum, ‘help inform’ the Canadian approach to negotiations and ‘help to ensure’ partners and stakeholders’ contributions would be ‘appropriately reflected’ in the final text.^56^

The Regional Engagement Series took place from 29 January 2024 to 12 February 2024.^57^ Further details confirmed that the sessions allowed the Government of Canada to share updates on the Agreement’s progress and provided a forum for participants to voice their opinions.^58^ Discussions centred around elements of the Agreement that required partners’ and stakeholders’ feedback and reminded participants that feedback should remain ‘practical, implementable and feasible’.^58^ Like the first engagement session in March 2023, an official report summarising the findings was released by the government of Canada, and the relevant findings are summarised below.

The government reported that participants suggested that the article on R&D required a better balance between public and private interests.^57^ Some, the government report points out, expressed the need to incentivise the private sector to build labs and research facilities in LMICs and expressed concern for public disclosure requirements that could discourage research.^57^ Some felt technology transfer, licensing and publicly funded research language were vague and required clearer definitions.^57^ Similarly, participants expressed the need for stronger language and accountability mechanisms in the sustainable production article to ensure products are sold at affordable prices.^57^ There was, according to the report, much focus on royalties, and while royalty-free licensing was advocated by some, the report also noted it was advanced that royalties are ‘an important incentive for the private sector’ and as such making them capped or limited could be an alternative approach.^57^ Discussions surrounding the article on technology transfer focused on maintaining incentives for innovation but not stripping the text of all legally binding obligations that ‘tip the scale’ too far in favour of the private sector.^57^ Participants saw gaps in the text, including a lack of ‘local, lived and Indigenous experience’, as well as a lack of understanding of what happens when voluntary technology transfer does not take place.^57^ Discussion of pathogen access and benefit sharing also centred around the contentious balancing of public and private interests. Some participants advocated for the retention of IP protections, while others suggested a ‘partnership approach’.^57^ Some stakeholders suggested benefit sharing may require a broader definition and there needs to be transparency in how ‘affordable’ is defined.^57^ Mention of South Africa’s disheartening experience with sharing Omicron data early was flagged, and some suggested looking at the UK’s approach to research as a model.^57^

Our analysis of the Canadian government’s report on the 2024 stakeholder consultations suggests subtle differences from the 2023 report. The subtext of the 2024 report seems, to us, to pay relatively more attention to industry stakeholder perspectives. It is not clear whether that is because there was greater industry participation in 2024 than in 2023, because the proposed treaty text had become more reflectively of industry positions, because the report was written with Canada’s negotiating positions in mind, or for some combination of these or other reasons.

#### March 2024 revisions

Following the eighth INB meeting in late February 2024, and in preparation for the ninth meeting in mid/late March 2024, a revised draft of the negotiating text of the Pandemic Agreement was released.^59^ By March 2024, it became clear that the Pandemic Agreement would no longer have the objective of ‘comprehensively and effectively addressing the systemic gaps and challenges that exist’.^53^ That phrase was deleted from the Agreement’s objective and scope.

The article on R&D changed significantly. Reference to pandemic-related R&D being aimed at ‘improving equitable access to and delivery of such products’ was removed entirely.^53^ However, the article did add a clause that ensures countries promote ‘equitable access to research knowledge’.^59^ Provisions on the sharing of publicly funded R&D were modified. The March text removed mention of ‘considering the extent of public funding provided’ when proposing that parties shall publish the terms of government-funded R&D agreements.^53^ Instead, it proposed parties develop national policies that include provisions that ‘promote timely and equitable global access’ to pandemic products and publish relevant terms of government-funded R&D agreements.^59^

The article on sustainable production also changed. The March text removed mention of encouraging ‘entities to grant non-exclusive, royalty-free licenses to manufactures’ and removed mention of encouraging R&D institutes to waive or manage royalties.^53^ Instead, the March draft focused on promoting and incentivising public and private investment and ‘promoting transparency of relevant unprotected information’.^59^

The central article on the transfer of technology and know-how saw several contextual and structural changes. The mention of ‘a set time frame’ to have developed mechanisms that promote technology transfer was removed.^53^ Moreover, the article added the option to ‘charge reasonable royalties’ on pandemic-related patents, rather than the ‘waiving’ of royalties referenced in the October text.^59^ The royalty-related provisions thus reflected the complete inverse of the original idea. Regarding the possibility of waivers of international IP obligations (ie, the TRIPS waiver issue), the March text was diluted, providing that parties ‘consider supporting’ time-bound waivers of IP during pandemic times, as opposed to parties committing to agree upon said waivers as was in the October text.^59^

#### March 2024 annotations on negotiating text

During the ninth INB meeting, WHO member states continued to debate provisions contained within the March 2024 negotiating text. An unofficial onscreen, annotated version of the negotiating text (as of 27 March 2024) was obtained and published by the media, providing further insight into the position of Canada and high-income allies on various IP provisions.^60^

Regarding R&D, Canada proposed to delete reference to parties promoting ‘equitable access to research knowledge’ and again add terms like ‘mutually agreed terms’.^60^ On sustainable production, Canada proposed adding a reference to ‘as needed’ and ‘to the extent possible’.^60^ Canada continued to propose adding a reference to ‘voluntary’ and/or ‘mutually agreed terms’ to multiple provisions under the article on technology transfer.^60^ The annotations also show Canada proposing to cap the provision on time-bound waivers by mandating parties to merely ‘consider supporting’ time-bound waivers and deleting reference to the purpose of the provision to ‘accelerate or scale up the manufacturing of pandemic-related products to the extent necessary to increase the availability and adequacy of affordable pandemic-related products’.^60^

Since deleting from earlier drafts any commitment of future support for new measures such as the TRIPS waiver, a replacement provision to at least recognise existing TRIPS flexibilities has been proposed instead. Such recognition would add nothing substantive to existing international trade law, but a subclause called ‘the peace clause’ was suggested as useful.^61^ The revised draft text included a proviso regarding TRIPS flexibilities, such as those embodied in the Doha Declaration, that parties to the Pandemic Agreement ‘shall fully respect the use thereof by others’.^60^ A more elaborate version of the peace clause proposed by LMICs would be even more explicit: ‘4*bis*. The Parties shall not challenge, or otherwise exercise any direct or indirect pressure on the Parties that undermines the right of WTO Members to use TRIPS flexibilities at any multilateral, regional, bilateral, judicial or diplomatic forum’.^60^ Canada and its high-income counterparts opposed either variation of this clause, suggesting the ideas be deleted from the draft.^60^

Canada also proposed deleting, in its entirety, a provision that would require countries to, as necessary and appropriate, review and update national legislation to allow for the full use of TRIPS flexibilities.^60^ Notably, this obligation complements the Parliamentary Committee’s 2022 recommendation that Canada immediately launch a public consultation on Canada’s Access to Medicines Regime and publish the findings within a year.^24^ Not only did the Canadian government not publish findings of a consultation within a year, as the committee recommended, but no public consultation was ever begun. That may explain Canada’s opposition to any provision in the Pandemic Agreement encouraging such domestic legislative review.

Apart from Canada’s position on weakening IP language, Canada in part continued to push forward the gender-inclusive agenda, requesting Article 16 on International Collaboration and Cooperation include specific mention of women in a provision that calls for equitable representation of decision makers.^60^ There was no mention of any effort to acknowledge or ameliorate the gender-related impacts of the IP provisions.

#### April 2024: a ‘proposal’ for the agreement

In April 2024, as the May 2024 negotiating deadline was imminent, the mainstream Canadian media reported that Canada’s push for weak language and vague aspirations was stalling negotiations.^62^

The article on research and development was significantly slimmed down. Canada’s push to remove reference to parties encouraging ‘equitable access to research knowledge’ was realised in the April text.^63^ Further, a standalone provision requiring member states to ‘support the transparent and public sharing of research inputs and outputs’ from government-funded R&D was removed.^63^ However, the draft retained a provision calling for member states to ‘ensure’ government funded R&D aimed at pandemic products includes ‘provisions that promote timely and equitable access to such products’, which may include ‘publication of relevant information on research inputs and outputs’ among other recommendations like mutually agreeable technology transfer or licensing.^63^

In the proposed agreement, Article 11 on technology transfer was merged with Article 10 on sustainable production. In effect, most of the ideas proposed and negotiated over the previous year were either abandoned or substantially scaled back. In the revamped Article 10, ‘mutually agreed terms’ are now the touchstone for the transfer of technology and know-how. The term was never mentioned in the previous March draft of the technology transfer article but was added to three separate provisions in the April text. A far cry from compulsory licensing, in the March and April drafts, even recipients of public funding are merely encouraged to forgo royalties or license patents ‘at reasonable royalties’ to developing country manufacturers to use technology and know-how, and even then, only encouraged to do so during a pandemic.

Mention of a time-bound waiver of international law, that is, the TRIPS waiver, had by this time almost disappeared entirely from the proposed text. The April 2024 draft was vague and weak, removing any mention of ‘time-bound waivers of intellectual property rights’ in the technology transfer article. As of April, the article simply called for states to ‘consider supporting, within the framework of relevant organisations, appropriate measures to accelerate or scale up the manufacturing of pandemic-related health products, to the extent necessary to increase the availability and adequacy of affordable pandemic-related health products during pandemics’.

#### May 2024: no final agreement

The resumed ninth meeting of the INB (29 April 2024–10 May 2024),was set to be the final round of negotiations before presenting a final agreement at the 77th World Health Assembly commencing 27 May 2024. However, recognising the progress made and the impending deadline approaching, the continued ninth meeting resumed from 20 to 24 May 2024.^64^ WHO members used the final push to deliver whatever they could, with the most useful outcome being a text showing exactly where progress was made and where it was not. While not a final text, the Agreement submitted to the World Health Assembly reflected this progress as of 24 May 2024. The updated draft included highlights showing initial agreement (green), convergence (yellow), no convergence (no colour) and divergent views (square brackets).

The article on R&D showed both agreement and debate. An initial agreement was reached on text calling on WHO member states to cooperate, collaborate and promote R&D initiatives while adding the phrases ‘as appropriate’ and ‘and in accordance with national and/or domestic law and policy’.^65^ The text was still to encourage the sharing of information and results through open science approaches; however, it also flagged that a definition for open science remained unsettled.^65^ Initial convergence was reached on a clause requiring parties, in accordance with domestic laws, to ‘support the transparent and public sharing of research results’; however, no consensus was reached on the provisions relating to the development and publication of policies for the timely and equitable access of pandemic products.^65^ The provisions on R&D remained stripped of any concrete obligations to fund R&D for equitable access to pandemic products.^66^

Little agreement was reached about the article on technology transfer, which continued to prove one of the most difficult to negotiate.^66^ One option was clarifying the definition of technology transfer itself and its association with voluntary and mutually agreed terms (VMAT), which had overwhelmed previous drafts. The association of technology transfer with VMAT has been controversial outside of the Pandemic Agreement negotiations, including in a recent global health resolution at the UN General Assembly.^66 67^ The article also presented various options for definitions for technology transfer itself, one of which would have clarified that technology transfer would be ‘consistent with relevant international norms regarding intellectual property’ and another claiming references to technology transfer would be ‘without prejudice to…flexibilities…under the provisions of the TRIPS Agreement’.^65^

There was no return to mention of time-bound ‘waivers’; rather, initial convergence was reached on the clause regarding ‘appropriate time-bound measures to accelerate or scale up the manufacturing of pandemic-related health products’.^65^ While a clause regarding respect for the use of the TRIPS agreement remained in the text, the use of the term TRIPS ‘flexibilities’ was unsettled. The clause also showed the proposed re-addition of language that members ‘shall not exercise any direct or indirect pressure…to discourage the use of such flexibilities’.^65^ Finally, the May 2024 text showed an initial convergence on a new clause that would require states to collaborate with the WHO to ‘identify, assess and, as appropriate, strengthen and /or] develop [multilateral] mechanisms that promote and facilitate the transfer of technology with a view to increasing access to pandemic-related products, particularly in developing countries, including through the pooling of intellectual property, know-how] and data and transparent, non-exclusive licensing’.^65^

#### July 2024: an extension and additional efforts

When no agreement was reached by the self-imposed May 2024 deadline, WHO members considered what to do next. In June 2024, the World Health Assembly decided to extend the mandate of the INB to produce a final text of the Agreement by the 78th World Health Assembly in 2025.^68^ At a July 2024 meeting of the INB,^69^ negotiators considered a proposed work plan and requested support in advance of the 11th INB meetings that were scheduled for September 2024. The INB sought a detailed programme of work, including intersectional work between September and November 2024 meetings. The INB also requested a schedule for interactive dialogues and outreach to provide balanced and diverse expertise, viewpoints and perspectives on two challenging issues of substance and two of form. On substance, the dialogues were to address ‘One Health’ (Article 5) and ‘Pathogen Access and Benefit Sharing’ (Article 12). On form, the dialogues were needed to assess the coherence between the amended International Health Regulations and the proposed Pandemic Agreement, as well as the ‘legal architecture’ of the proposed agreement.

##### September and November 2024: options for a new legal architecture

The information document requested at the 10th INB meeting in July was produced prior to the 11th meeting in September.^70^ It confirmed the intent was for a legally binding instrument that would contain both legally binding and non-binding elements. The document outlined possible ways in which coherence between a proposed Pandemic Agreement and the International Health Regulations might be achieved, referencing principles of international law and the Vienna Convention on the Law of Treaties. Notably, the document outlined considerations regarding ‘annexes’ and ‘protocols’. Annexes are integral and therefore part of the same legal instrument, while protocols are separate legal instruments. Going forward, information on the legal architecture of the proposed Pandemic Agreement is likely to be important. That is because this information suggests multiple pathways forward, many of which are not mutually exclusive.

Meanwhile, the text continued to progress at the 11th meeting of the INB in September 2024. The unofficial draft text, reflecting progress up until 16 September 2024, showed some increased consensus about the technology transfer provisions, increasing from four to six agreed-upon clauses.^71^ This included agreement on the promotion/incentivisation of technology transfer and know-how for pandemic-related health products through mechanisms like licensing.^71^ Furthermore, regarding R&D, there was still no agreement, and rather divergent views, on the development and publication of policies for the timely and equitable access of pandemic products.^71^

Initial convergence was reached on including the term ‘open science’ in the clause about promoting research collaboration and access to research; however, as of September 2024, it is unclear what the definition will be. While there is still no agreement, and rather divergent views, on the development and publication of policies for the timely and equitable access of pandemic products, there has been initial agreement now calling on states to ‘support the rapid and transparent publication of clinical trial protocols and other research results related to the implementation of this agreement’, in accordance with domestic laws, policies and international standards. An initial agreement has been reached on the clause surrounding transparency of information, including that transparency be limited to information and raw materials ‘not subject to protection’ under relevant domestic and international law. In other words, trade secrecy or other IP rights trump transparency.

Finally, regarding technology transfer, a definition has not been agreed upon, but a proposition has been made in a footnote to Article 11:

Transfer of technology is understood to mean non-coercive transfer and on mutually agreed terms. This understanding is without prejudice to other measures that parties may take pursuant to their domestic and/or national legislation, provided that such measures are consistent with their relevant international obligations regarding intellectual property.

If and when the definition has been agreed upon, it is written that there has been initial agreement that each party shall, as appropriate, ‘*Promote and otherwise facilitate or incentivise transfer of technology and know-how for pandemic-related health products, in particular for the benefit of developing countries, [and for technologies that have received public/government funding for their development in line with Article 9] through measures which may include, inter alia, licensing, capacity building, relationship facilitating, incentives or conditions linked to research and development, procurement or other funding and regulatory policy measures*’. As indicated by the role of square brackets, there are still divergent views on whether publicly funded technology will be mentioned in this clause.

It is clear from the latest text that the phrase, ‘on mutually agreed terms’, has become the touchstone of technology transfer. With this, and reference only to existing national legislation consistent with international obligations, the reality sinks in that the world is no further ahead on this issue than before the pandemic.

On the topic of time-bound waivers, agreement remains elusive with various forms of watered-down language proliferating. As a means of summarising the evolving discourse around IP, open science, technology transfer and related matters, our accompanying annex (online supplemental file 1) shows how, as of the publication of this article, the world went from a potential agreement to support time-bound waivers of IP to a weak expression of agreement to ‘consider supporting… appropriate measures…to the extent necessary…during pandemics’ to a mere reference to ‘mutually agreed terms’ and compliance with national law and existing international obligations.

## Conclusion

The process of negotiating new international health law—from the WTO TRIPS waiver discussions to the idea for a WHO Pandemic Agreement—was fraught with difficulty. In this article, we have shown through textual and contextual analysis how provisions impacting IP, open science, technology transfer, pathogen access and benefit sharing and more were negotiated. By focusing on Canada’s role and position, the study adds depth and specificity to the analysis of global negotiations while also offering insights relevant to the global health community. Seeing how forum shifting and negotiation strategies lead to compromises in international health law and policymaking enables a better understanding of emerging legal rules and their likely practical impacts, facilitates more effective national implementation of international law and supports further efforts to promote equity in global health.

## Supplementary material

10.1136/bmjph-2024-001467 online supplemental file 1

## Data Availability

No data are available.
