# Supplementary material for: Negotiation of new international health law on intellectual property, technology transfer, open science and pathogen access and benefit sharing: a textual and contextual analysis
Source: BMJ Public Health. 2024 Dec 17;2(2):e001467. doi: 10.1136/bmjph-2024-001467 (PMC12581020; doi:10.1136/bmjph-2024-001467)
Supplement: online supplemental file 1 [file bmjph-2-2-s001.pdf]

Annex: Provision on the Waiver of States' International Legal Obligations Regarding Intellectual Property Rights

Date	Document	Article	Text
25 November 2022	**Conceptual zero draft** for the consideration of the Intergovernmental Negotiating Body at its third meeting (A/INB/3/3)	Article 7. Access to technology: promoting sustainable and equitably distributed production and transfer of technology and know-how	2. Towards this end, each Party [shall]/[should]: . . . (iv) measures to support time-bound waivers of protection of intellectual property rights that are a barrier to manufacturing of pandemic response products during pandemics
1 February 2023	**Zero draft** of the WHO CA+ for the consideration of the Intergovernmental Negotiating Body at its fourth meeting (A/INB/4/3)	Article 7. Access to technology: promoting sustainable and equitably distributed production and transfer of technology and know-how	In the event of a pandemic, the Parties: (a) will take appropriate measures to support time-bound waivers of intellectual property rights that can accelerate or scale up manufacturing of pandemic-related products during a pandemic, to the extent necessary to increase the availability and adequacy of affordable pandemic-related products;
2 June 2023	**Bureau's text** of the WHO convention, agreement or other international instrument on pandemic prevention, preparedness and response (WHO CA+) (A/INB/5/6)	Article 11. Co-development and transfer of technology and know-how	Option 11.A<br>5. In the event of a pandemic, the Parties shall: (a) take appropriate measures to support time-bound waivers of intellectual property rights that can accelerate or scale up the manufacturing of pandemic-related products during a pandemic, to the extent necessary to increase the

			availability and adequacy of affordable pandemic-related products;<br><br>Option 11.B<br><br>Option A for 5(e): suspend the application of intellectual property rights, through time-bound waivers, in order to facilitate the scaling-up, production, manufacture and supply of the products that are especially meant for a pandemic. Neither Party shall challenge these measures based on any international obligations that the Party suspending the obligation may have
30 October 2023	**Proposal for negotiating** text of the WHO Pandemic Agreement (A/INB/7/3)	Article 11. Transfer of technology and know-how	3. During pandemics, each Party shall, in addition to the undertakings in paragraph 2 of this Article: (a) commit to agree upon, within the framework of relevant institutions, time-bound waivers of intellectual property rights to accelerate or scale up the manufacturing of pandemic-related products to the extent necessary to increase the availability and adequacy of affordable pandemic related products;
13 March 2024	**Revised draft of the negotiating text** of the WHO Pandemic Agreement (A/INB/9/3)	Article 11. Transfer of technology and know-how	3. During pandemics, in addition to the undertakings in paragraph 1 of this Article, each Party shall: …<br><br>(b) consider supporting, within the framework of relevant institutions, time-bound waivers of intellectual property rights to accelerate or scale up the manufacturing of pandemic-related products to the extent necessary to increase the availability and adequacy of affordable pandemic-related products.

22 April 2024	**Proposal for the WHO Pandemic Agreement**<br><br>(A/INB/9R/3)	Article 11. Transfer of technology and know-how for the production of pandemic related health products	"3. Consider supporting, within the framework of relevant organizations, appropriate measures to accelerate or scale up the manufacturing of pandemic related health products, to the extent necessary to increase the availability and adequacy of affordable pandemic-related health products during pandemics."
24 May 2024	**Proposal for the WHO Pandemic Agreement**<br><br>Draft text reflecting progress up to Friday 24 May at 12:00 CEST<br><br>(A/77/10 Annex)	Article 11. Transfer of technology and know-how for the production of pandemic-related health products	"3. Each Party shall [consider supporting], within the existing framework of relevant international and regional organizations, appropriate time-bound measures to accelerate or scale up the manufacturing of pandemic-related health products, to the extent necessary to increase the availability, accessibility and affordability of pandemic-related health products during pandemic emergencies.
16 September 2024	**Proposal for the WHO Pandemic Agreement**<br><br>Draft text reflecting progress up to Monday 16 September at 17:30 CEST<br><br>(A/77/10 Annex)	Article: Article 11. Transfer of technology and know-how for the production of pandemic-related health products	"3. Each Party shall, within the [existing] framework of relevant international and regional organizations, [consider supporting] [adopts] [consider adopting] [review and consider] [endeavour to undertake] [support, as] appropriate, time-bound measures to accelerate or scale up the manufacturing of pandemic-related health products, to the extent necessary to increase the availability, accessibility and affordability of pandemic-related health products during pandemic emergencies."
